# Coordination of Leaf Development Across Developmental Axes

**DOI:** 10.3390/plants8100433

**Published:** 2019-10-22

**Authors:** James W. Satterlee, Michael J. Scanlon

**Affiliations:** School of Integrative Plant Science, Cornell University, Ithaca, NY 14853, USA; jws429@cornell.edu

**Keywords:** leaf, developmental patterning, transcription factors, plant hormones, differentiation

## Abstract

Leaves are initiated as lateral outgrowths from shoot apical meristems throughout the vegetative life of the plant. To achieve proper developmental patterning, cell-type specification and growth must occur in an organized fashion along the proximodistal (base-to-tip), mediolateral (central-to-edge), and adaxial–abaxial (top-bottom) axes of the developing leaf. Early studies of mutants with defects in patterning along multiple leaf axes suggested that patterning must be coordinated across developmental axes. Decades later, we now recognize that a highly complex and interconnected transcriptional network of patterning genes and hormones underlies leaf development. Here, we review the molecular genetic mechanisms by which leaf development is coordinated across leaf axes. Such coordination likely plays an important role in ensuring the reproducible phenotypic outcomes of leaf morphogenesis.

## 1. Introduction

**Figure 1:**
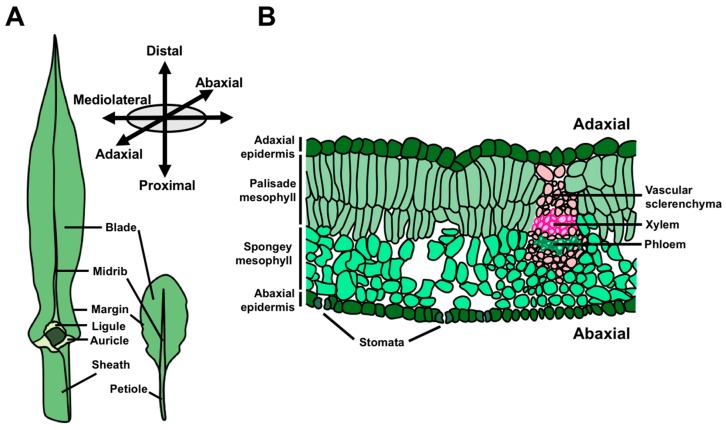
Leaf anatomy and growth axes. (**A**) Leaves from maize, a monocot, and the two closely related eudicots, *Cardamine hirsuta* and *Arabidopsis thaliana*, highlighting major features of leaf anatomy. (**B**) Cross-section of a mature eudicot leaf illustrating the characteristic features of the adaxial and abaxial leaf domains.

The morphology of angiosperm leaves is extraordinarily diverse, and is a key component of natural variation in plant architecture. Nonetheless, all leaves share two ontogenetic features in common; they are (1) derived from the shoot apical meristem (SAM) and (2) asymmetrical from their inception [1]. The SAM is a stem cell reservoir at the growing tip(s) of the plant that ultimately supplies cells for all the above-ground organs. In this way, the SAM accounts for the continuous development of vegetative structures far beyond embryogenesis, which comprises a critical, strategic difference between plant and animal development. A leaf transitions from its origins as a small primordium to its mature form through the establishment and maintenance of three developmental axes upon which growth and differentiation proceed (Figure 1A,B) [2,3]. The leaf proximodistal axis (i.e., base-to-tip) is defined by the polarized growth of leaf initials away from the shoot, and later becomes elaborated by the establishment of proximal and distal cell and tissue types. For example, in many eudicot leaves, such as those of *Arabidopsis*, the leaf is subdivided into the proximal petiole and distal lamina. Leaf development also involves specialization of the upper and lower leaf surfaces, defining an adaxial–abaxial (top-bottom) axis of asymmetry. At its inception, the leaf primordium possesses inherent asymmetry along this axis due to the proximity of the adaxial leaf surface to the SAM relative to the abaxial leaf surface [1,4]. This early asymmetry drives the differentiation of diverging cell and tissue fates in the adaxial and abaxial domains of the leaf. Common anatomical differences between these domains include the formation of adaxial xylem and abaxial phloem, as well as differences in epidermal and mesophyll cell morphology. Finally, leaves grow to form a widened, flattened lamina from a marginal domain at the juxtaposition of the adaxial and abaxial leaf faces, thereby defining the mediolateral axis of the leaf. Development along the mediolateral axis is associated with the positioning of the medial leaf midvein and the proliferative transverse outgrowth of cells to form the leaf lamina, or blade.

A host of transcriptional regulators underlie the establishment and maintenance of the leaf developmental axes, often making use of inhibitory interactions to specify opposing cell fates. A classic example of this type of regulatory logic is seen in the context of the conserved antagonistic relationship between Class I KNOTTED-LIKE HOMEOBOX (KNOX), ASYMMETRIC LEAVES1 (AS1), and ASYMMETRIC LEAVES2 (AS2) transcription factors [5,6,7,8,9,10,11,12,13,14]. *Class I KNOX* genes are expressed in meristematic tissue and promote indeterminate growth, while AS1 and AS2 repress *KNOX,* an important first step in the transition from indeterminate to determinate cell identity along the proximodistal axis. Inhibitory interactions between adaxial and abaxial patterning regulators are also well-described. Along this axis, the adaxial cell fate-promoting HD-ZIP Class III (HD-ZIP III) transcription factors are repressed by abaxial cell fate-promoting KANADI (KAN) transcription factors and the HD-ZIPIII-targeting miR165/166 microRNAs [15,16,17,18,19,20]. In a parallel pathway, transcripts of the abaxial cell fate-promoting ETTIN/AUXIN RESPONSE FACTOR3 (ETT/ARF3) and ARF4 transcription factors are targeted by small-interfering RNAs (ta-siARFs) expressed in the adaxial domain [21,22,23]. Together, these mutually inhibitory interactions precisely specify and maintain the boundaries between the adaxial and abaxial faces of the leaf.

The reproducible phenotypic outcomes of leaf development may in part be ensured by multiple layers of redundancy built into the underlying genetic patterning network. Not only do multiple pathways act in parallel to regulate the same patterning processes, but gene duplication has expanded the number of redundantly acting pattering genes, potentially allowing for patterning robustness in the face of network perturbation or noise [24,25,26,27]. For example, the *Arabidopsis* genome contains four copies of the abaxially expressed *KAN* transcription-factor-encoding genes, which redundantly promote abaxial cell fate [28,29]. While genetic redundancy may allow for stable and predictable developmental outcomes along a given leaf axis, leaves develop simultaneously along multiple axes such that mechanisms controlling three-dimensional development must be synchronized to some extent. In the context of vertebrate limb bud development, evidence has emerged for molecular coordination across organ axes. For example, the transcription factors HAND2 and GLI3R, which promote anterior and posterior cell fate, respectively, have been shown to directly cross-regulate the expression of genes important for proximodistal patterning [30,31,32]. In plants, a model wherein adaxial–abaxial patterning establishes outgrowth of both the mediolateral and proximodistal leaf axes was proposed almost 25 years ago [33]. Here, we review several decades of developmental genetics research that test and extend this classical model of the mechanisms whereby leaves initiate from the SAM and undergo coordinated development along three axes. In general, coordinating factors sit at the top of complex gene regulatory networks, and work to organize and control the expression of well-characterized patterning modules. Their context-specific functions are frequently mediated by physical interaction and tightly regulated feedbacks with other patterning factors, to yield consistent morphological outcomes. Key patterning genes mentioned in this article are listed in Table 1.

## 2. Coordination of the Proximodistal and Adaxial–Abaxial Leaf Axes

### 2.1. ASYMMETRIC LEAVES1 and ASYMMETRIC LEAVES2

The *Antirrhinum phantastica* (*phan*) mutant was the first mutant described with impaired development along multiple leaf axes. Leaves of *phan* mutants exhibit a temperature-sensitive loss of adaxial–abaxial polarity, as well as ectopic *KNOX* gene expression in the leaf, indicating a role for *PHAN* in coordinating pattern formation along both the adaxial–abaxial and proximodistal axes [33,34]. The subsequent cloning of *phan* identified a loss-of-function mutation in a MYB-domain transcription factor-encoding gene as responsible for the phenotype [34]. *PHAN* orthologs are described in maize, *Arabidopsis*, tobacco, tomato, and pea [10,12,13,35,36,37,38,39]. Despite the high degree of sequence conservation among the *PHAN* orthologs, the *phan* phenotype differs across species. In maize, loss-of-function of the *PHAN* ortholog *ROUGH SHEATH2 (RS2)* yields leaves with ectopic *KNOX* expression, resulting in the intrusion of proximal tissue types, such as the auricle and ligule, into distal portions of the leaf blade [40,41,42]. This suggests that *KNOX* genes may act as proximal patterning factors in maize leaves and is consistent with detection of KN1 protein in the developing leaf base of wild-type leaves [43]. However, unlike *phan* mutants, leaf adaxial–abaxial polarity is retained in *rs2* maize mutants [14]. In contrast, mutations in the *PHAN* orthologs of eudicots show proximodistal patterning defects, as well as varying degrees of adaxial–abaxial polarity defects, similar to the originally reported *phan* phenotype in *Antirrhinum*. In *Arabidopsis*, loss of function of the *PHAN* ortholog *ASYMMETRIC LEAVES1* (*AS1*) results in leaves that are asymmetric, shortened along the proximodistal axis, and exhibit intrusion of petiole-like epidermal cells into the leaf lamina [10,44,45]. Similar to maize, these features are accompanied by ectopic Class I *KNOX* gene expression in the leaf [44,46]. Additionally, subtle defects in adaxial–abaxial polarity are observed [46,47]. More extreme patterning defects are observed in tobacco *phan* loss-of-function mutants [40]. Leaves lose much of their leaf blades and adaxial–abaxial patterning is perturbed throughout the petiole [48]. Phenotypic severity of *phan* is reduced under gibberellic acid (GA) treatment, a hormone that promotes differentiation and is biosynthetically inhibited by *KNOX* activity in undifferentiated tissues [49,50]. Finally, in pea *phan* mutants, proximal stipules are displaced distally and leaflet adaxial–abaxial polarity is perturbed, whereas in tomato *phan* mutants, the entire leaf is severely abaxialized [41,42,43]. Despite the differences in the *phan* phenotypes across taxa, there is a conserved role for *PHAN* in the downregulation of *KNOX* gene expression, and, to varying degrees, the regulation of adaxial–abaxial patterning in eudicots. Studies of *phan* phenotypes, therefore, set the stage for further investigation into cross-axes coordination of development in leaves.

**Figure 2:**
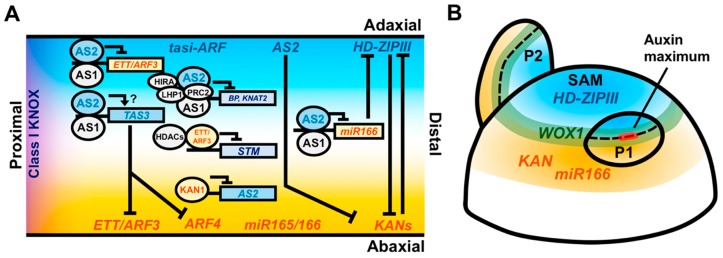
Coordination of adaxial–abaxial and proximodistal leaf patterning factors. (**A**) Longitudinal model cross-section of the leaf with adaxial factors labeled in cyan, abaxial factors labeled in orange, and proximal factors labeled in purple. *AS2* and *HD-ZIPIII* transcripts accumulate in the adaxial domain of the leaf, while *ETT/ARF3*, *ARF4*, miR165/166, and *KAN* transcripts accumulate in the abaxial domain. miR165/166 and the mature *ta-siARF* RNAs are capable of moving intercellularly and repressing *HD-ZIPIII* and *ETT/ARF3* postranscriptionally. *HD-ZIPIII* and *KAN* genetically antagonize one another and *AS2* can genetically repress *KAN*. The AS1–AS2 complex recruits the HIRA protein and the PRC2/LHP1 complex to repress expression of Class I *KNOX* genes and also directly binds and represses the expression of *ETT/ARF3* and *miR166* in the adaxial portion of the leaf. AS1–AS2 binds *TAS3*, which encodes the ta-siARF precursor transcript, possibly as a protective mechanism to exclude *TAS3*-antagonizing transcription factors from accessing their promoter binding sites. The abaxial factor ETT/ARF3 directly represses the expression of the Class I *KNOX* gene *STM* by recruiting HDACs. KAN restricts *AS2* expression to the adaxial domain via a direct repressive interaction. (**B**) Radially organized expression domains of patterning factors within the meristem may prepattern the adaxial–abaxial axis of the leaf. *HD-ZIPIII* genes are expressed centrally while *KAN* and *miR166* are expressed peripherally. *WOX1* expression occurs at the juxtaposition of these domains. The auxin response is restricted to the boundary region where auxin promotes outgrowth of the leaf primordium, retaining the prepatterned domains of gene expression during the establishment of the proximodistal leaf axis.

Shortly after the cloning and characterization of *AS1* in *Arabidopsis*, a second mutant was reported with perturbed leaf symmetry, resulting in the formation of highly asymmetric leaf lobes, as well as overgrowth of the adaxial leaf surface [11,51,52]. This mutant, named *asymmetric leaves2* (*as2*), harbors a loss-of-function mutation in a gene encoding a LATERAL ORGAN BOUNDARIES (LOB)-domain transcription factor. *AS2* is expressed uniformly throughout early-stage leaf primordia before becoming restricted to the adaxial domain, and similarly to *AS1*, negatively regulates the expression of Class I *KNOX* genes. In maize, the *AS2* homolog *INDETERMINATE GAMETOPHYTE1* (*IG1*) confers similar patterns of ectopic *KNOX* expression, as well as adaxial–abaxial polarity defects [53]. Overexpression of *AS2* in *Arabidopsis* leads to leaf adaxialization [54]. Therefore, *AS2,* like *AS1*, links patterning along both the proximodistal and adaxial–abaxial leaf axes (Figure 2A). In fact, AS2 interacts physically with AS1, forming a complex that directly regulates the expression of *BREVIPEDICELLUS* (*BP*), an *Arabidopsis* Class I *KNOX* gene [55]. The repressive activity of the AS1–AS2 complex is mediated by its interaction with repressive chromatin remodeling factors, such as HIRA, in both *Arabidopsis* and maize [56]. In *Arabidopsis*, the AS1–AS2 complex can also recruit the POLYCOMB REPRESSIVE COMPLEX2 (PRC2) and LIKE HETEROCHROMATIN PROTEIN1 (LHP1) to repress *KNOX* gene expression [57,58]. Consistent with the role of the AS1–AS2 complex in the repression of proximal gene expression programs through downregulation of *KNOX* gene expression, the defects in midrib patterning, ectopic stipules, and leaf lobing—but not the defects in adaxial–abaxial patterning—are reduced by loss of class I *KNOX* gene function in the *as1 as2* mutant background [59]. Treatment with GA also rescues the proximodistal patterning defects seen in the *as1 as2* double mutants, consistent with previous work in tobacco [40].

Meanwhile, AS1 and AS2 coordinately regulate adaxial–abaxial patterning by repressing a distinct set of genes promoting abaxial cell fate. AS1–AS2 directly binds the abaxial identity gene *ETT/ARF3*, and represses its expression in the adaxial domain of the leaf via the recruitment of DNA methylating enzymes (Figure 2A) [60]. AS2 also binds *ARF3*, along with *miR166a* and *TAS3A*, two small-RNA encoding genes implicated in adaxial–abaxial patterning and *AS2* is, in turn, repressed in the abaxial domain via direct binding by KAN1 (Figure 2A) [61,62]. AS2 represses *KAN1* but it is unknown whether this interaction is direct. Taken together, AS1 and AS2 function in a highly conserved pathway to coordinately repress *KNOX* gene expression. The role of AS1 in leaf adaxial patterning in eudicots may largely be ascribed to its interaction with AS2, which regulates adaxial–abaxial patterning in both eudicots and monocots. The lack of adaxial–abaxial polarity defects in monocot *as1* mutants could, therefore, reflect modifications in AS1–AS2 complex function and/or genetic redundancy (reviewed in [63]). Overall, the AS1–AS2 complex offers an example of developmental coordination across leaf axes through regulation of multiple downstream patterning modules and interactions with a diverse array of regulatory cofactors.

### 2.2. Auxin Controls Proximodistal and Adaxial–Abaxial Axis Formation

The plant hormone auxin also has key roles in coordinating the development of the proximodistal and mediolateral leaf axes, both downstream and upstream of key patterning factors. Polarized transport of auxin to foci at the flanks of the SAM predicts the site of leaf initiation and is critical for the initiation of the proximodistal leaf axis [64,65]. Auxin accumulation is driven by the polarization of PIN1 (PIN-FORMED1) auxin efflux carriers in the L1 layer of the meristem. Through an as yet undetermined pathway, the accumulation of auxin represses the expression of *Class I KNOX* gene expression at the site leaf primordium initiation, a necessary first step in proximodistal axis formation [66,67]. Interestingly, auxin application to the SAM is only able to promote primordial outgrowth at the SAM periphery, suggesting that an auxin response competency is required for the initiation of the proximodistal axis. This region of competency correlates with juxtaposed domains of cells expressing the *HD-ZIPIII*, *KAN,* and *miR166a* adaxial–abaxial patterning genes (Figure 2B) [4,5,68,69]. The expression of these genes within the SAM may reflect a prepattern that organizes the location of auxin-induced proximodistal outgrowth while simultaneously specifying the adaxial and abaxial domains of the initiating primordium. The homeobox transcription factor WUSCHEL (WUS), which is expressed in the SAM central zone and functions to maintain a stable population of meristematic stem cells, directly represses the expression of *KAN1/2*, suggesting a mechanism by which this prepattern is maintained [70]. How these adaxial–abaxial patterning factors mediate the restriction of auxin responsiveness is currently unknown. Nevertheless, it necessitates the coordination of proximodistal outgrowth with a predefined adaxial–abaxial prepattern, which likely contributes to stable patterning outcomes during leaf initiation.

Auxin may also play a role in coordinating the development of the proximodistal and adaxial–abaxial axes beyond the leaf initiation stage. High levels of auxin signaling are associated with the distal tips of the leaf and the developing vasculature [66,71] Meanwhile, treatment of tomato leaf primordia with exogenous auxin or the disruption of auxin transport and signaling is capable of abaxializing the leaf, suggesting that auxin functions to maintain the abaxial leaf domain [72]. However, contradictory evidence suggests a role for auxin in adaxial cell fate acquisition, as auxin promotes the expression of the *HD-ZIPIII* gene *REV* [68]. In fact, more recent analysis revealed no overall differences in auxin perception between the adaxial and abaxial leaf surfaces, arguing against a role for differential auxin accumulation along the adaxial–abaxial leaf axis [73]. Nonetheless, the observation of auxin-induced leaf abaxialization remains unexplained [72]. It is possible that polarized expression of ARFs, which mediate the transcriptional response to auxin, may control the differential effects of auxin within the abaxial and adaxial leaf domains *downstream* of auxin perception [21,74]. These genes may also serve as integrators of auxin-regulated adaxial–abaxial and proximodistal patterning in the leaf, since *ETT/ARF3* directly binds and represses *STM* via the recruitment of histone deacetylases (Figure 2A) [75]. Further dissection of the role of ARFs in mediating the outcomes of auxin signaling during leaf development is clearly warranted and may reveal their activity in coordinating morphogenesis across developmental axes.

## 3. The Coordination of Adaxial–Abaxial and Mediolateral Patterning Underlies the Predictions of the Waites–Hudson Model

Waites and Hudson’s study of the *Antirrhinum phan* mutant first revealed the intimate relationship between the establishment of adaxial–abaxial polarity and the ability of the leaf to grow wide along the mediolateral axis [33,39]. This laid the foundation for the Waites–Hudson model, which predicts that adaxial–abaxial polarity is a prerequisite for mediolateral growth of the leaf. The study of numerous polarity mutants in both monocots and eudicots supports this model; when adaxial or abaxial cell fates are lost, leaves fail to grow wide and instead form radialized filamentous structures. In some cases, regions of blade outgrowth can occur between juxtaposed domains of adaxial or abaxial tissue, suggesting that mediolateral outgrowth can occur where these boundaries adjoin one another [17,28,29,76,77]. However, the presence of adaxial–abaxial polarity alone is not sufficient to drive mediolateral outgrowth. For example, the maize mutant *ragged seedling2* (*rgd2*) possesses normal adaxial–abaxial patterning but often fails to form a blade [78].

### 3.1. PRESSED FLOWER and WOX1 Coordinate Mediolateral Outgrowth with Adaxial–Abaxial Patterning

Two important regulators of mediolateral development acting downstream of adaxial–abaxial patterning are the homeobox genes *PRESSED FLOWER* (*PRS*) and *WUSCHEL-LIKE HOMEOBOX1* (*WOX1)*. Loss of *PRS* function is associated with missing lateral sepals and stamens in the flower and deletion of lateral stipules at the leaf base [79,80]. The maize orthologs of *PRS, NARROW SHEATH1* (*NS1*) and *NARROW SHEATH1* (*NS2*), also have roles in patterning leaf outgrowth from the leaf margin; the width of the sheath and proximal leaf blade are severely reduced in *ns1 ns2* double mutants [80,81]. Orthologs of *NS1* and *NS2* have been cloned in other monocot species, including rice (*NARROW LEAF1 2/3* [*NAL2/3*]) and barley (*NARROW LEAF DWARF1* [*NLD1*]), and yield comparable phenotypes when disrupted, although defects in margin patterning extend well into the leaf blade in these species [82,83]. While grass genomes lack a *WOX1* paralog, *WOX1* orthologs are present in the genomes of *Arabidopsis,* tobacco (*LAMINA1* [*LAM1*]), petunia (*MAEWEST [MAW]*), and *Medicago* (*STENEFOLIA* [*STF*]) and have conserved roles in promoting the mediolateral outgrowth of the leaf blade [84,85,86]. The *wox1 prs* double mutant exhibits an even more extreme reduction in leaf width, suggesting that the two genes work together to promote mediolateral leaf development. Not only do these mutants have narrow leaves, but the adaxial–abaxial polarity of the epidermis and mesophyll is perturbed at the leaf margins, strongly indicating a role for these genes in axial coordination [85,87].

Prior to leaf initiation, the prepatterned adaxial–abaxial polarity within the meristem may organize the mediolateral domain of the primordium in which *WOX1/PRS* are expressed. Fluorescent reporter studies identified a circumferential band of *WOX1* expression around the meristem at the boundary between *HD-ZIPIII* and *KAN* expression domains (Figure 2B) [68]. Subsequently, feedback regulation of *WOX1*/*PRS* is seen at later stages of leaf development. In *kan1 kan2* double mutants, *WOX1* and *PRS* are misexpressed in the abaxial domain of the leaf, suggesting that the *KAN* genes may function to negatively regulate the expression of *WOX1*/*PRS* [87]. Misregulation of *WOX1/PRS* may, therefore, explain the ectopic formation of abaxial margin-like outgrowths that occur in higher order *kan* mutants. *AS2* also appears to negatively regulate the expression of *WOX1/PRS* in the adaxial domain of the leaf. *AS1* orthologs in tomato and tobacco influence the extent of the adaxial domain in leaves and thereby, the positions where mediolateral growth can occur [41,48]. One possibility is that the AS1–AS2 complex contributes to the restriction of the *WOX1/PRS* domain in the leaf. Together, these interactions could help to restrict *WOX1/PRS* to the margin domain of the leaf, thereby rendering mediolateral outgrowth dependent on proper adaxial–abaxial patterning and lending a mechanistic basis to the predictions made by Waites and Hudson.

**Figure 3:**
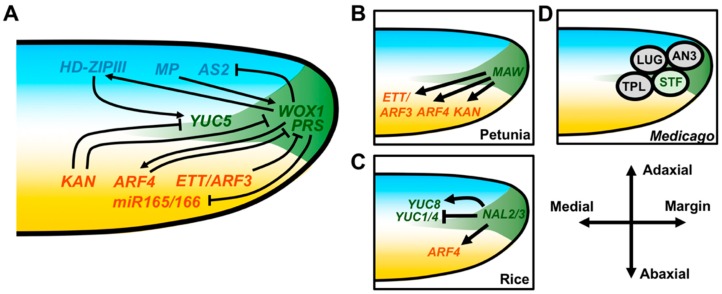
Genetic interactions between marginal and adaxial–abaxial patterning genes at the leaf margin. (**A**) *WOX1/PRS* inhibits the expression of the adaxial patterning gene *AS2* and the abaxial patterning genes miR165/166. Inhibition of miR165/166 indirectly promotes expression of *HD-ZIPIII* genes in the adaxial domain but may also do so via other direct/indirect mechanisms. *KAN*, *ETT/ARF3* and *ARF4* can repress *WOX1/PRS* and *WOX1/PRS* can promote *ARF4*. Auxin biosynthesis at the margin is promoted by expression of the auxin biosynthesis gene *YUC5*, which is inhibited by *KAN* and promoted by *HD-ZIPIII*. (B-D) WOX1 and PRS activity in other plant species. (**B**) The petunia homolog of *WOX1*, *MAW*, promotes the expression of *ETT/ARF3*, *ARF4*, and *KAN* homologs. (**C**) In rice, the PRS orthologs *NAL2* and *NAL3* repress the expression of *ETT/ARF3* and *ARF4* homologs as well as the auxin biosynthesis genes *YUC1* and *YUC4*. *YUC8* is instead upregulated by *NAL2/3*. (**D**) The *Medicago* WOX1 protein STF can physically interact with the TPL and LUG corepressor proteins but also with the transcriptional activator AN3.

Given the adaxial–abaxial patterning defects in *wox1 prs* mutants, *WOX1* and *PRS* are likely involved in feedback regulation of adaxial–abaxial patterning factors at later stages of development. Among the adaxial patterning factors, *WOX1/PRS* promote the expression of *HD-ZIPIII* genes in the developing leaf margins, likely in part through inhibition of miR165/166 (Figure 3A) [84] *WOX1/PRS* also repress the expression of *AS2*, restricting its expression to the adaxial domain of the leaf margin [87,88]. Meanwhile, *WOX1/PRS* influence the expression of abaxial-specific genes as well. For example, *ARF4* expression is weakly promoted by *WOX1* in *Arabidopsis* while *ETT/ARF3, ARF4,* and *KAN* paralogs in petunia are all upregulated by *MAW* (Figure 3A,B) [85]. *ARF4* expression is also promoted by *NAL2/3* in rice (Figure 3C) [82]. The means by which *WOX1/PRS* modulate expression of these adaxial–abaxial patterning genes remains unresolved, as well as the functional importance of these regulatory interactions. *WOX* genes appear to act in a majority of cases as transcriptional repressors, and STF is known to interact with the corepressor proteins LEUNIG (LUG) and TOPLESS (TPL) but also with AINTEGUMENTA3 (AN3), a transcriptional activator important for regulating cell proliferation (Figure 3D) [89,90,91,92]. Therefore, it is probable that both transcriptional activation and repressive functions of WOX1/PRS in the developing leaf margin maintain a stable boundary between the adaxial and abaxial leaf domains, thereby coordinating the patterning of the adaxial–abaxial and mediolateral leaf axes.

### 3.2. Auxin Interconnects Adaxial–Abaxial and Mediolateral Patterning

Naturally, auxin also has a role in coordinating development along the mediolateral and adaxial–abaxial leaf axes. Local auxin biosynthesis, transport, and signaling all have a role in patterning the leaf margin and are under the direct transcriptional control of the opposing adaxial–abaxial patterning factors REV and KAN1 [93,94,95,96]. Auxin biosynthesis is necessary for marginal patterning, as higher-order loss-of-function mutations in the *Arabidopsis YUCCA* (*YUC*) auxin biosynthesis genes lead to defective margin development and REV directly promotes *YUC5* expression, whereas KAN1 directly represses it [97]. In rice, *NAL2/3* repress *YUC1* and *YUC4* but promote *YUC8* expression [82]. Together, these data suggest that localized auxin biosynthesis may act downstream of adaxial–abaxial and/or mediolateral patterning to promote outgrowth at the margins (Figure 3A) [93,94,95,96].

Transport of auxin within the developing leaf is also crucial for proper development of the mediolateral and adaxial–abaxial leaf axes. In tomato, loss of PIN1 function results in occasional leaf radialization and disruption of auxin transport in lateral domains of the incipient leaf primordium [72]. Incisions separating the incipient leaf primordium from the SAM can also induce leaf radialization, and new data suggest that this phenomenon may be the consequence of perturbed auxin transport into the leaf [68,98,99].

Despite the apparent importance of spatiotemporal regulation of auxin transport and accumulation for leaf development, levels of auxin perception appear remarkably uniform across the adaxial–abaxial axis of the leaf, indicating potential polarization of the auxin response machinery [73]. ARF proteins mediate the transcriptional response to auxin and fall into two clades: type-A ARFs that possess transcriptional activation properties, and type-B ARFs, which act as transcriptional repressors [100,101,102]. The abaxial identity-promoting genes *ARF3/ETT* and *ARF4* are both repressive Type-B ARFs. Other ARFs implicated in the control of adaxial cell fate include MONOPTEROS (MP), a Type-A ARF expressed in the adaxial domain of the leaf [103,104]. These abaxially and adaxially expressed ARFs appear to converge on the transcriptional regulation of *WOX1* (Figure 3A). *MP* promotes the expression of *WOX1*, whereas *ARF3/ETT* and *ARF4* repress it, consistent with their functional classifications [104]. Therefore, the complementary expression patterns of these Type-A and Type-B ARFs may translate auxin stimuli into instructions to pattern both the adaxial and abaxial faces of the leaf, thereby stably restricting the expression domain of *WOX1* at the leaf margin. MP lacking the AUX/IAA-binding domain only conditions adaxial–abaxial patterning defects when expressed in the adaxial leaf domain, hinting that other auxin signaling components necessary for MP activity may be polarized along the adaxial–abaxial axis [103,105].

## 4. Control of Mediolateral Patterning along the Proximodistal Axis

### 4.1. Coordinating Indeterminate Growth and Growth Cessation across Axes

**Figure 4:**
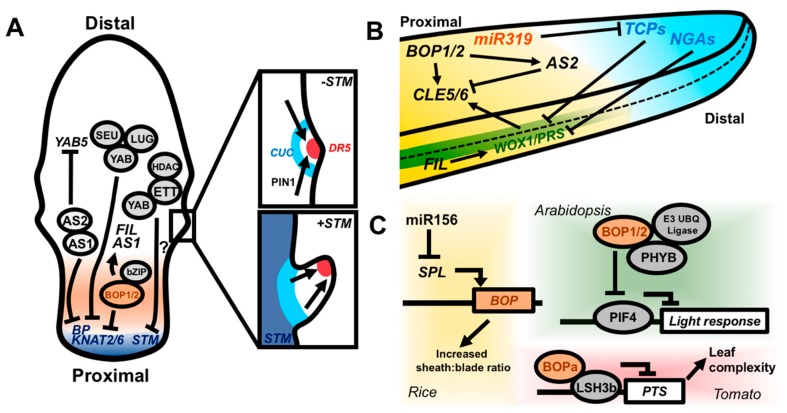
Molecular coordination of the proximodistal and mediolateral leaf axes. (**A**) The Class I *KNOX* genes *BP* and *KNAT2/6* are repressed by the AS1–AS2 complex, as well as by YAB transcription factors in complex with the corepressors SEU and LUG. The AS1–AS2 complex is, in turn, able to repress *YAB5*. YABs and ETT both repress *STM* expression and they can physically interact, potentially forming a repressive complex. BOP1/2 also repress *BP* and *KNAT2/6* and promote expression of *FIL* and *AS1*. If Class I *KNOX* genes are not repressed in the leaf, growth is prolonged, which ultimately leads to the formation of leaflets via PIN1, auxin, and *CUC2* activity (insets). Local auxin accumulation (indicated by expression of the DR5 promoter) driven by PIN1-mediated transport, drives margin outgrowth, while *CUC* genes inhibit growth in the surrounding sinus region. (**B**) Side-on view of a leaf illustrating the cross-regulation of proximodistal and mediolateral patterning factors in the context of the growth arrest front. The TCP and NGA genes promote cell differentiation and growth cessation in the distal portion of the leaf and are antagonized by proximally expressed miR319. TCPs and NGAs coordinately repress the expression of *WOX1/PRS* to allow for growth cessation at the margin. *WOX1/PRS* promote the proximal expression of *CLE5/6*, which affect leaf shape. *BOP1/2* and *AS2* also converge to promote and inhibit the expression of *CLE5/6*, respectively. BOP1/2 directly promote *AS2* expression. *FIL* may promote mediolateral outgrowth by promoting *WOX1* expression. (**C**) Genetic pathways by which BOPs may integrate internal and external cues to regulate patterning processes. In rice, miR166 represses *SPL* expression, which, in turn, inhibits *BOP* expression in the juvenile phase. Low *BOP* expression confers an increased sheath:blade ratio in juvenile leaves. Silencing of *miR156* expression in adult leaves leads to increased BOP expression, yielding reduced blade:sheath ratios. BOP1/2 likely also function in light signaling. *Arabidopsis* BOP1/2 interact with PHYB and an E3 ubiquitin ligase complex to degrade PIF4 and attenuate light responsive gene expression. BOPa in tomato interacts with the light response regulator LSH3b and LSH3b represses *PTS* expression with downstream KNOX-mediated promotion of leaf complexity.

Leaf width can vary extensively along the proximodistal axis, suggesting that regulators of proximodistal patterning may influence mediolateral development. In eudicots, *KNOX* genes regulate patterning processes at the leaf margins. For example, the loss of *BP, KNAT2,* and *KNAT6* function in the *as1* mutant background can rescue many of the patterning defects triggered by ectopic Class I *KNOX* gene expression: the reduced petiole lengths and lobed margins conditioned by *as1* are rescued [10,45,46,57]. In plants with compound leaves, *KNOX* genes have the additional role of promoting leaflet development at the leaf margins. For example, the *STM* gene is necessary for the formation of leaflets in the *Arabidopsis* relative *Cardamine hirsuta*, and overexpression of the *STM* ortholog *KN1* is sufficient to yield highly dissected compound leaves in tomato [106,107,108]. *STM* acts by prolonging indeterminate, proliferative growth in the leaves of *C. hirsuta*, which is critical for the formation of leaflets [108,109] (Figure 4A). Leaflets are, in turn, patterned by the activity of auxin and *CUP-SHAPED COTYLEDON* (*CUC*) genes at the leaf margins. Marginal auxin maxima are generated by polarized auxin transport, thereby promoting leaflet outgrowth, whereas *CUC* expression is restricted to surrounding areas where it restricts growth [110,111]. This same mechanism is used to pattern the small serrations on the margins of *Arabidopsis* leaves, yet the lack of prolonged proliferative growth driven in part by *STM* in *C. hirsuta* prevents serrations from becoming fully fledged leaflets (Figure 4A). Unlike *BP* and *KNAT2*/6, *STM* is not subject to negative regulation by *AS1,* although *as1* loss-of-function mutants in tomato have reduced numbers of leaflets. *AS1* appears to affect leaflet number via its control of adaxial identity; leaves that fail to properly establish juxtaposed adaxial–abaxial leaf domains produce fewer leaflets in more distal positions [41,48]. Therefore, the regulation of cell indeterminacy by *KNOX* and *AS1* along the proximodistal axis governs patterning outcomes at the margins.

Growth cessation is also tightly coordinated between the proximodistal and mediolateral leaf axes. As the leaf matures, a wave of reduced cell-proliferation migrates basipetally across the leaf. Members of the TEOSINTE BRANCHED1/CINCINNATA/PROLIFERATING CELL FACTOR (TCP) transcription factor family, along with the NGATHA (NGA) transcription factors, regulate the movement of this arrest front, thereby promoting the cessation of growth in the leaf [112,113,114,115]. The proximally expressed microRNA *miR319* counters TCP activity by post-transcriptionally silencing *TCP* mRNAs (Figure 4B). As growth ceases along both the proximodistal and mediolateral axes, it is reasonable to infer that these inhibitors of cell proliferation may also act to control the expression of mediolateral patterning genes. In fact, the combined overexpression of *miR319a* and knockout of *NGA1,2,3* and *4* induces prolonged expression of *WOX1/PRS*, leading to severe overproliferation of the leaf margin (Figure 4B) [116]. This places *WOX1* and *PRS* downstream of the TCP-NGA-miR319 proximodistal patterning module and provides a potential mechanism by which growth cessation along both the proximodistal and mediolateral axes is synchronized, yielding properly proportioned leaves.

### 4.2. YABBY Genes Regulate Proximodistal and Mediolateral Leaf Development

Members of the plant-specific YABBY (YAB) family of transcription factors are also important coordinators of mediolateral and proximodistal leaf development. Originally, *YAB* genes were linked to the control of abaxial cell identity in the leaf [117,118,119,120]. In *Arabidopsis*, *FIL* and other *YABs* are expressed in the abaxial domain of the leaf primordium; however, in other plant species, the spatial expression patterns of *YAB* genes diverge from the situation in *Arabidopsis*. Namely, maize *YAB* genes are expressed adaxially, whereas in rice, *YAB* genes exhibit no apparent polarity in developing leaf primordia [18,76,121,122]. Furthermore, overexpression of the abaxializing factor *KAN2* in *Arabidopsis* does not condition ectopic expression of *FIL*, suggesting that the spatial regulation of *FIL* expression is in some sense independent of leaf adaxial–abaxial polarity [76]. Instead, *YAB* genes appear to at least in part regulate the growth of the leaf mediolateral axis. In support of this, the ectopic laminar outgrowths found on the abaxial side of *kan1 kan2 kan3* mutants fail to develop in the *yab3* background [76]. Furthermore, quadruple *yab* mutants show severely perturbed expression of margin patterning factors, yet adaxial–abaxial patterning genes are only mildly affected [123]. The *PRS/WOX1* patterning module may have a conserved role in regulating the expression of *YAB* genes. In *Arabidopsis*, *WOX1* promotes the expression of *FIL* and, in rice, *NAL2/3* positively and negatively regulates the expression of multiple *YAB* genes [82,87,124]. Therefore, *YAB* genes likely act, to an extent, downstream of mediolateral patterning processes at the leaf margin.

Meanwhile, alongside their roles in mediolateral patterning, *YAB* genes antagonize the activity of proximal patterning factors. The *KNOX* genes *KNAT2, BP,* and *KNAT6* are upregulated in the *yab3 fil* mutant background, and *yab3 fil* partially suppresses the *stm* mutant phenotype [125]. Consistent with these findings, ectopic meristems form on the adaxial leaf face in *yab3 fil* mutants. Transcriptomic analysis of leaves from higher-order *yab* mutants identified expression of *WUS* in leaf tissue, again indicating that meristem and/or axillary meristem (i.e., proximal) gene expression programs are reactivated in the leaf [123]. In *Arabidopsis*, FIL, YAB3, and YAB5 repress gene expression by forming a complex with the transcriptional repressor proteins LEUNIG and SEUSS (Figure 4A) [126]. Genetic disruption of this complex is associated with larger and more disorganized SAMs, suggesting that this complex may act non-cell autonomously to repress meristematic gene expression. Effects on SAM size are also observed in loss-of-function mutants of two maize *YAB* genes, *DROOPING LEAF1/2 (DRL1/2);* however, the effect is reversed compared to *Arabidopsis*, as *drl1 drl2* mutant meristems are smaller than wild-type [121]. In rice, *YAB1* works in a negative regulatory circuit with the hormone GA, which promotes leaf growth and differentiation. *YAB1* is upregulated by GA and, in turn, represses expression of the GA biosynthesis gene *GA3OX2* [122]. A lack of GA homeostasis in monocot *yab* mutants may explain the smaller SAM phenotypes of *drl1/2* maize mutants, as meristematic cell identity is in part dependent on GA biosynthesis repression via KN1 [49,50]. Clearly, if this regulatory network exists in eudicots, its importance for regulating SAM size is diminished. Finally, YABs can also interact with ETT/ARF3 in vitro, raising the possibility that this interaction may contribute to the known downregulation of Class I *KNOX* genes by ETT/ARF3 via their interactions with histone deacetylases (Figure 4A) [75]. Together, these findings place *YAB* genes as key regulators of mediolateral and proximodistal patterning in leaves, yet the precise roles of *YAB* function is rendered ambiguous owing to the species-specific differences in phenotypes. For example, the characterized *YAB* genes of maize and rice control differentiation of the leaf midrib: no defects in mediolateral or adaxial-abaxial polarity are observed [121,127]. A better understanding of *YAB* gene function in the context of proximodistal and mediolateral patterning will likely emerge as reverse genetics overcomes the high levels of genetic redundancy in the *YAB* family.

### 4.3. BLADE-ON-PETIOLE Genes Integrate Developmental Patterning with Environmental Sensing

Genes that coordinate the development of multiple leaf axes simultaneously may also be poised to mediate broad developmental changes in response to endogenous or exogenous cues. Such roles are emerging for the *BLADE ON PETIOLE* (*BOP*) genes. *BOPs* encode BTB/POZ domain transcription factors that harbor an ankyrin repeat. In *Arabidopsis*, *bop1* and *bop2* mutants develop ectopic outgrowths of blade tissue along the petiole [128,129,130]. Thus, *BOP* genes may restrict mediolateral outgrowth to the appropriate position along the proximodistal axis of the leaf [131]. The *BOP* genes repress the expression of the Class I *KNOX* genes *BP, KNAT2,* and *KNAT6* in *Arabidopsis* [128]. Furthermore, in cauline leaves and floral lateral organs the *BOP* genes are required for the development of the proximal abscission zone by regulating the expression of *ATH1* (*ARABIDOPSIS THALIANA HOMEOBOX1*) [132,133]. BOP1/2 possess transcriptional activation activity, and may repress Class I *KNOX* gene expression via their direct upregulation of *AS2* [134,135]. BOP1/2 can also physically interact with bZip proteins, an interaction involved in controlling *ATH1* expression [136]. Meanwhile, BOP1/2 may regulate leaf width by controlling the expression of mediolateral patterning factors. BOP1/2 promote the expression of *FIL*, which could stimulate the mediolateral expansion of the leaf blade at specific positions along the proximodistal axis [134]. In addition, BOP1/2 promote the expression of two proximally expressed CLE (CLAVATA-LIKE EMBRYO SURROUNDING REGION) peptide-encoding genes that are simultaneously upregulated by *WOX1* and repressed by *AS2* [137]. CLEs typically have intercellular signaling roles and bind to leucine-rich receptor-like kinases. In *Arabidopsis*, *cle5/6* mutants exhibit subtle changes in leaf width and petiole angle. BOP1/2 may, in part, regulate the mediolateral development of the leaf by converging with WOX1 and AS2 on *CLE5*/*6* regulation (Figure 4B).

Recently, BOPs have also been identified as orchestrating developmental responses to endogenous and external cues. For example, *BOP* gene function in rice intersects with the vegetative phase change pathway. Rice contains three *BOP*-like genes and higher-order mutant combinations lead to progressively reduced blade:sheath ratios, with this effect becoming more pronounced as leaves transition from juvenile to adult stages [138]. Indeed, the juvenile leaf 1 has a much higher level of *BOP* expression than do subsequent leaves and is almost entirely composed of sheath tissue. Phase-specific regulation of *BOP* expression is driven by the highly conserved *miR156*-*SQUAMOSA PROMOTER BINDING LIKE* (*SPL*) pathway in which reduction in *miR156* expression relieves the repression of *SPL*, thereby promoting the juvenile-to-adult phase transition (Figure 4C) [139]. *BOP* genes are likely downstream of this pathway but, it is unknown whether BOPs are direct targets of *SPL*. BOPs also mediate the developmental outcomes of light signaling. PHYTOCHROME B physically interacts with both BOP1 and BOP2 in *Arabidopsis*, and BOP2 can mediate the degradation of the light-response transcriptional regulator PHYTOCHROME INTERACTING FACTOR4 (PIF4) through a physical interaction with an E3 ubiquitin ligase complex under red light (Figure 4C) [140]. BOPs also interact with the LIGHT-DEPENDENT SHORT HYPOCOTYLS3b (LSH3b) protein of tomato [141]. In *Arabidopsis*, *LSH* genes promote cell growth in response to light and, in tomato, LSH3b binds and negatively regulates *PETROSELINUM*, which enhances leaf complexity through its posttranscriptional modification of KNOX activity [141,142,143]. Therefore, BOP control of development along the proximodistal and mediolateral axes may act downstream of both internal and external cues to coordinately pattern the leaf.

## 5. Conclusions

Plants are capable of producing consistently patterned leaves throughout their lifespan, a feat that requires highly robust control of underlying genetic patterning factors. One way that such robustness may be achieved is through the close coordination of factors patterning the three growth axes of leaves, a prediction realized by the work of Waites and Hudson almost twenty five years ago. Here, we have described several genetic and hormonal factors that simultaneously regulate developmental patterning along multiple leaf axes simultaneously. Such control mechanisms likely ensure that growth along these axes proceeds in a synchronized and organized fashion. Perhaps most striking is the highly redundant repression of Class I *KNOX* genes by factors regulating development along all three axes. Clearly, *KNOX* repression is an essential and tightly regulated developmental program of the leaf. Additionally, coordinating factors frequently cross-regulate multiple patterning modules, as well as interact with a host of co-regulatory proteins to carry out their functions. Looking forward, advances in live imaging methods will allow for enhanced analysis of leaf development and its associated spatiotemporal gene expression patterns in real time. This, coupled with rapdily advancing genomics technologies, will provide a major boon to understanding the complex and interwoven leaf axial patterning pathways. Through such work, we will come closer to understanding the remarkable consistency of leaf patterning in three dimensions.

## Figures and Tables

**Table 1:** Key genes mentioned in the text, their relevant homologs in other species, and the leaf axes they pattern.

Arabidopsis Gene Name	Homologs (Discussed in This Review)	Leaf Axes^1^
*SHOOTMERISTEMLESS* (*STM*)	*KNOTTED1* (*KN1*; maize)	PD
*BREVIPEDICELLUS*(*BP*), *KNAT2*, *KNAT6*		PD
*ASYMMETRIC LEAVES1* (AS1)	*PHANTASTICA* (*PHAN; Antirrhinum*)*, ROUGH SHEATH2 (RS2; maize)*	PD,ML, AA
*ASYMMETRIC LEAVES2* (*AS2*)	*INDETERMINATE GAMETOPHYTE* (*IG1*; maize)	PD,AA
*PHABULOSA* (*PHB*), REVOLUTA (REV), *PHAVOLUTA* (*PHV*)	*ROLLED LEAF1 (rld1*; maize)	AA
*ETTIN/AUXIN RESPONSE FACTOR3* (*ETT/ARF3*), *AUXIN RESPONSE FACTOR4*	*ARF3a* (maize)	AA,ML
*MONOPTEROS* (*MP*)		AA,ML
miR165/miR166	miR165/miR166 (maize)	AA
*TAS3A*	*TAS3A* (maize)	AA
*KANADI1/2/3* (*KAN*)	*MILKWEED POD1* *(mwp1*; maize)	AA
*PIN-FORMED1* (*PIN1*)		PD
*PRESSED FLOWER* (*PRS, WOX3*)	*NARROW SHEATH1/2* (*NS1/2*; maize), *NARROW LEAF2/3* (*NAL2/3*; rice), *NARROW LEAF DWARF* (*NLD1*; barley)	ML,AA
*WUSCHEL-LIKE HOMEOBOX1* (*WOX1*)	*LAMINA1* (*LAM1*; tobacco), *MAEWEST* (*MAW*; petunia), *STENEFOLIA* (*STF*; *Medicago*)	ML,AA
*CUP-SHAPED COTYLEDON2* (*CUC2*)		ML
*TEOSINTE BRANCHED1/CINCINNATA/PROLIFERATING CELL FACTOR (TCP)*		PD
*NGATHA* (*NGA*)		PD
*miR319*		PD
*FILAMENTOUS FLOWER (FIL), YABBY3 (YAB3), YABBY5 (YAB5)*	*DROOPING LEAF1/2* (*DRL1/2*, maize), *YABBY1* (*YAB1*, rice)	PD,ML
*BLADE ON PETIOLE1/2* (*BOP1/2*)		PD,ML

^1^ PD: proximodistal, ML: mediolateral, AA: adaxial–abaxial.
